# Cutting uncertain stock and vehicle routing in a sustainability forestry harvesting problem

**DOI:** 10.1007/s11750-022-00623-7

**Published:** 2022-03-12

**Authors:** Adejuyigbe O. Fajemisin, Steven D. Prestwich, Laura Climent

**Affiliations:** 1 Amsterdam Business School, University of Amsterdam, Amsterdam, The Netherlands; 2 Insight Centre for Data Analytics, School of Computer Science and IT, University College Cork, Cork, Ireland; 3 Departamento de Ingeniería Informática, Universidad Autónoma de Madrid, Madrid, Spain

**Keywords:** Multiple Stock Size Cutting Stock Problem, Uncertain stock, Vehicle routing, Sustainable forestry harvesting

## Abstract

Sustainable forest management is concerned with the management of forests according to the principles of sustainable development. As a contribution to the field, this paper combines the Vehicle Routing Problem (VRP) (in which the vehicles are harvesters) with the Multiple Stock Size Cutting Stock Problem under uncertainty (in which the stock is logs). We present an Integer Linear Program that dynamically combines the cutting of the uncertain stock with vehicle routing, and uses it to address real-life problems. In experiments on real data from the forestry harvesting industry, we show that it outperforms a commonly used metaheuristic algorithm.

## Introduction

In this paper, we deal with a *Multiple Stock Size Cutting Stock Problem* (MSSCSP) under uncertainty. The original *Cutting Stock Problem* (CSP) described in Gilmore and Gomory (1961) is a well-known NP-hard optimisation problem in Operations Research. This problem is of interest in many industries because of the perennial problem of dividing large pieces of stock material into smaller pieces to meet customers’ demand for certain products types, while minimising waste. In the classic CSP, all stock items have the same known measurements, which makes it easier to compute the possible *cutting instructions*. However, in MSSCSPs (CSP topology from Wäscher et al. (2007)), the stock may have different sizes.

The first contribution of this paper consists in the formalisation of the forestry harvesting problem as a MSSCSP. Forestry harvesting is a real-life sustainability application. These applications aim to avoid the depletion of natural resources, and at the same time allow the quality of life of modern societies to increase. In forestry harvesting (as with many sustainability real applications), there is an additional complication: log sizes are estimated, and these estimates differ (sometimes significantly) from real log sizes. This causes two difficulties: (i) cutting instructions for stock items with unknown measurements cannot be computed; (ii) even if we could use similar patterns to the sampled stock, the amounts obtained from cutting the real stock will differ from those expected. Dealing with such difficulties represents another contribution of the paper.

In previous work (Climent et al. 2016a), the forestry harvesting problem was classified according to a CSP typology (from Dyckhoff (1990)) as $*/V/D/R$ where $*$ means any dimensionality, *V* means that the total amount of items in stock is sufficient to accommodate all the demanded products (hence, only some of the stock will be cut), *D* means that all large pieces (stock items) are different (in terms of shape) and *R* indicates many products of few different types are demanded. The feature *V* (any demand can be fulfilled) requires that the stock items to be cut need to be selected.

Other sustainability applications have similar characteristics: a heterogeneous assortment of large pieces of possibly unknown measurements. All these applications can be included in population harvesting [see Getz and Haight (1989)], as it is impossible to measure all possible members of the population; moreover, the sizes of the members change with time. The forestry harvesting problem is an example of such a case due to the extremely large number of trees of varied sizes, combined with the continuous growth of the trees. Due to the impossibility of measuring all the logs in the forest, only a small sample of them is measured and these are the only data available.

The two difficulties mentioned above make the problem much harder to solve. Difficulty (i) implies that it is impossible to compute the cutting instructions of all the stock items (because not all the stock is known). This requires a more complex way of determining the best cutting instructions. This process is explained in detail in Sect. 3, but we mention here that the process involves cutting simulations over the sampled stock. In the literature, there is a metaheuristic algorithm that performs many simulations in a bounded time, and provides a solution to the cutting instructions used in the best simulation. However, we solve the problem with a different approach: via Monte Carlo simulation [see Mooney (1997)] and an Integer Linear Program (ILP).

Due to difficulty (ii) (cut amounts differ from expected amounts), we tackle the MSSCSP dynamically by re-computing solutions: first computing the difference between expected and obtained amounts, and second updating the “targeted” amounts as the harvesting process progresses. Our second contribution is an ILP that dynamically combines the MSSCSP with the vehicle routing problem to decide not only which stock to cut and how to cut it, but also the path that the cutting machines should follow for optimising the resources.

The importance of including uncertainty and dynamism to realistically model real-life applications was emphasised in Climent et al. (2014). Real applications have a lack of information (uncertainty) and some of the associated data of the original specifications evolve with time (dynamism). In the literature, there are ILP approaches that are “static”: they only compute a single solution for the MSSCSPs and do not consider that the expected and obtained amounts may be different, which is unrealistic in real-world applications. Even if the sampled stock is a good representation of the whole population (which is not always the case), the cutting results will not be the same as those in the simulation phase. This is the motivation for developing our technique which re-calculates targeted amounts after cutting each real stock subset. The re-calculated amounts are then used to obtain new solutions for the remaining stock.

For this reason, we present a novel ILP that combines the VRP with a particularly difficult CSP that dynamically reacts to uncertainty in the data. To the best of our knowledge, there are no dynamic approaches in the literature that use ILP for addressing this combination of problem features. Only the metaheuristic approach previously mentioned addresses the same problem as this paper. For this reason, we evaluate our algorithm and we compare it with such an approach using real data provided by the industry. We show that, given the same computation time, our technique leads to greater resource preservation than the metaheuristic approach.

## Literature review

In this section, we provide the literature review associated with the variant of MSSCSP with uncertainty in Sect. 2.1. A literature review of the VRP in sustainability problems, with a focus on agriculture, is provided in Sect. 2.2.

### MSSCSP

There are several approaches in the literature for solving MSSCSPs using either exact methods such as those in Belov and Scheithauer (2002) and Alves and de Carvalho (2008), heuristic methods as in Poldi and Arenales (2009), or a combination of the two as in Hemmelmayr et al. (2012). For MSSCSPs related to our problem, some methods use ILP but do not address the uncertainty of the MSSCSPs. However, there are also metaheuristic approaches that deal with uncertainty.

The importance of considering uncertainties regarding the stock measurements, and dealing with them dynamically, is evidenced by Hung et al. (2012). The authors deal with uncertain amounts of product types. They present an approach that first computes the possible patterns for each stock size, then starts the on-line process of selecting a pattern for each stock piece to cut (after detecting its size). However, in our MSSCSP, the uncertainty comes from the real measurements of the stock, as only a sample of them is available. This fact precludes the use of the technique in Hung et al. (2012) (precomputing patterns and on-line selecting them) because we cannot compute the patterns of raw material for which the measurements are unknown.

In Dems et al. (2017), the authors also present an ILP approach for solving a similar MSSCSP but without dynamism. They also use a different algorithm, which uses a priority list of product types instead of priority vectors, to generate the cutting instructions. This is a disadvantage because most of the cutting machines in the industry work with an algorithm that cuts the materials according to the priority vector. While the stock items are sometimes cut to maximise the market value of the products, at other times the goal is to satisfy the demand for the products (which sometimes comes at the expense of their market values). Furthermore, Dems et al. (2017) only handle 16 cutting instructions, while we can handle several hundred. The other improvement of our technique over Dems et al. (2017) and also Prestwich et al. (2015) is that we address the dynamic on-line cutting process: we re-calculate targeted amounts and re-compute solutions at the end of each cutting stage. Moreover, these two previous works do not compare themselves with a previous and popular metaheuristic approach while we do (see Sect. 6).

Climent et al. (2016c) present a different proactive approach, which is not applicable only to the CSP but to many large-scale combinatorial optimisation problems. It takes into account the uncertainty in the amounts of products that are expected to be obtained. However, to apply this technique, it is necessary to know the range of values for each of the amounts expected. Our experience with industries that work in sustainability applications tells us that these data might be unknown, so we have, therefore, developed a reactive approach, which does not need to take this information into account.

Another way of addressing a CSP (without considering dynamism) is to use some form of column generation. In this approach, the CSP is first solved using a subset of the cutting instructions for a fixed number of raw material pieces with similar measurements. New patterns are then introduced by solving an auxiliary optimisation problem, and the process is repeated iteratively until completion. The column generation approach was originally applied to the standard CSP in Gilmore and Gomory (1961, 1963), and was used in the forestry problem by Eng et al. (1986), Laroze and Greber (1997) and Mendoza and Bare (1986). However, this technique cannot be applied to the real-life application treated in this paper since, as previously mentioned, for this problem, it is not possible to pre-compute the patterns of the stock that has not been sampled.

Besides the ILP approaches mentioned earlier, a metaheuristic approach was also developed earlier for MSSCSPs [see Murphy et al. 2004; Climent et al. 2016b]. The metaheuristic approach is a Simulated Annealing Like Algorithm (SALA) called Threshold Accepting Algorithm (TA) (Dueck and Scheuer 1990). It iteratively generates new priority vectors (by making local changes) that are mapped by a Dynamic Programming (DP) algorithm (Bellman and Dreyfus 2015; Anderson et al. 2015) into cutting instructions. Its objective is to reduce the difference between the percentages of product types obtained by a pattern, and the percentages demanded. In Murphy et al. (2006), a dynamic version of the metaheuristic is presented, in which the targeted demands are re-calculated after cutting each stock subset. However, their algorithm randomly selects the next stock subset to be cut, while we select the most suitable stock subsets to be cut and their order (by combining the CSP with the VRP). We believe that this is one of the key factors for the improved performance of our technique.

### VRP in agriculture

The classic VRP is well known, and at its simplest, it consists in finding the optimal route (or set of routes) from one or more depots through a set of destination nodes, subject to certain constraints (Laporte 1992). A practical example of this is the routing of delivery vehicles through locations in a city to deliver packages to customers. There are several variations of the VRP: for example in cases where a return to the depot is not required, the VRP is known as an open VRP (MirHassani and Abolghasemi 2011). Also, each vehicle may or may not have a limit on its package-carrying capacity: Capacitated (Cordeau et al. 2007) and Uncapacitated (Eksioglu et al. 2009) VRPs respectively. A good reference on the many vehicle routing problem variants and applications is given in Toth and Vigo (2014). A taxonomic review of the VRP, including its variants and methods of solution, is also given in Braekers et al. (2016).

The VRP also finds application in agriculture and has been used, for example, in scheduling the collection and transportation of livestock (Oppen and Løkketangen 2008; Sigurd et al. 2004), in managing the operation of farm machinery in separated fields in Bochtis (2008), for the planning of autonomous tractor operations in Bochtis et al. (2009, 2008), and for determining the optimal routes for combining the harvesters in Ali et al. (2009). A dedicated classification of agricultural field operations concerning the VRP is given in Bochtis and Sørensen (2009, 2010). In Briot et al. (2015), the authors try to minimise the working time (including travel time) of grape harvesters, which gives the problem some resemblance to routing problems. Basnet et al. (2006), similar to Bochtis (2008), use the VRP for farm-to-farm path determination for scheduling crop harvesting in multi-farm operations. We would like to highlight that the above-mentioned papers do not combine the VRP with the MSSCSP.

In this paper, we combine the VRP and the MSSCSP, which is similar to the routing problem with stock loading constraints reviewed in Iori and Martello (2010), as cutting and packing problems are analogous. In our problem, however, there are several differences. First, unlike in the standard routing and loading problems, e.g. Doerner et al. (2007); Fuellerer et al. (2010); Junqueira et al. (2013); Miao et al. (2012), we are concerned with the routing of the cutting machines rather than the vehicles that transport the cut stock. Our main concern is to select the right priority vector to be used by the cutting machines in each block. Next, we make the decision of which blocks should be cut and in which order, by selecting the best possible route (the one that minimises the transport costs). Besides, this problem represents a real-life application and typically in these contexts there is unknown data about some of the parameters of the problem Climent et al. (2016c). Specifically, in the problem treated in this paper, there is uncertainty regarding the expected stock sizes and the real stock sizes, while the above-mentioned papers do not consider this uncertainty.

## Problem description and formalisation

In this section, we describe and formalise the problem treated in the paper, by formalising it as an uncertain Multiple Stock Size Cutting Stock Problem (MSSCSP). We introduce its special characteristics and provide a mathematical formulation.

### General problem description

**Fig. 1:**
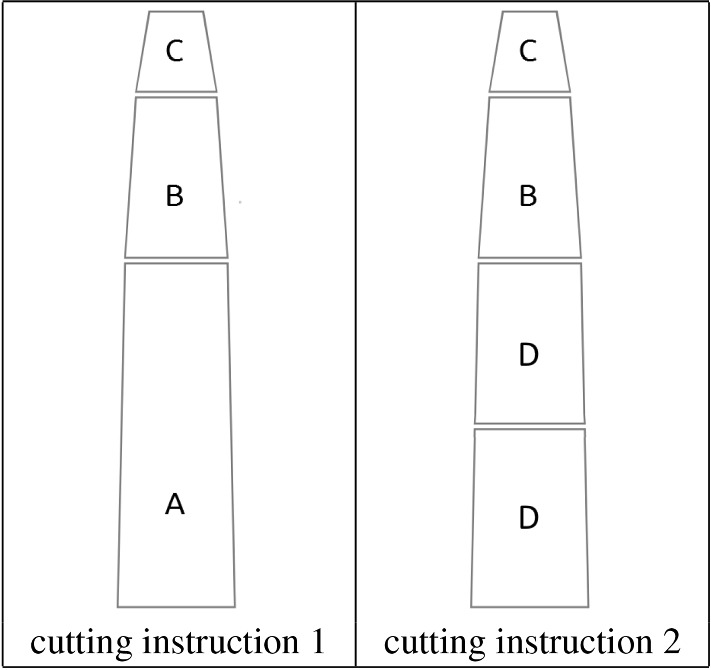
Two different cutting instructions applied to the same log

As previously mentioned, MSSCSPs have raw material pieces of different measurements which we can cut at will. Recall that for forestry harvesting the pieces of material are the logs of the trees and the cutting machines are the harvesters. In Fig. 1, we can observe a log of a tree cut in two different ways. Each cutting instruction produces different amounts of the log products of types A, B, C and D. To generalise to other domains (see population harvesting problems in Getz and Haight (1989)), we use the term “raw material pieces” (or stock) and cutting machines. In the variant of MSSCSP that we are dealing with, we only know a limited number of raw material pieces (typically several hundred) each with its measurements. However, they only represent a sample of the real raw material pieces (typically several thousand). The measurements of the whole set of pieces are only known when the real cutting of the pieces is performed. We recall the impossibility of measuring all the members of the population because of time limits.

Since only a fraction of the stock is known in advance, an approach for deciding how to cut the piece of stock when the machine is performing the real cutting is necessary (by which time the machine has access to its measurements). Moreover, deciding how to cut each piece must be based on constraints on the total product demands and the overall objective of using the minimum stock possible. Therefore, it is necessary to decide the cutting instruction for a single piece based on the previous cuts of the rest of the pieces (which have already produced certain amounts of products). Waste must also be minimised. For these reasons, both the literature and industry use a cutting algorithm that decides how to optimally cut each piece of stock (here each log) and reduce the waste. This algorithm is described in the next subsection.

### Cutting algorithm for individual pieces of stock

The algorithm for cutting an individual piece takes as input a vector of priorities of the products demanded by the customers. It is a vector of continuous variables, with each component of the vector corresponding to the monetary value of each of the products in which the stock is to be cut into. For example in Fig. 1, type A of the log-products could have a revenue of 10€$/\text{m}^3$, while type C of log-product could have a revenue 3€$/\text{m}^3$ (note that type A is bigger than type B). The objective of this algorithm is to maximise the total value of the cutting of a single piece of stock based on the priority vector. Note that since the waste has a monetary value of zero, it tends to be minimised.

The cutting algorithm is implemented with DP, which allows us to compute the optimal cutting instruction for a given piece of stock. DP is an approach that enables the solution of complex problems by dividing them into a collection of simpler sub-problems. To do this, the sub-problems must be sequential and independent. The problem of cutting a raw material piece satisfies these properties since it is a recursive one (i.e. maximise the benefit by cutting the first product and then maximising the benefit by cutting the remaining raw material piece).

**Fig. 2:**
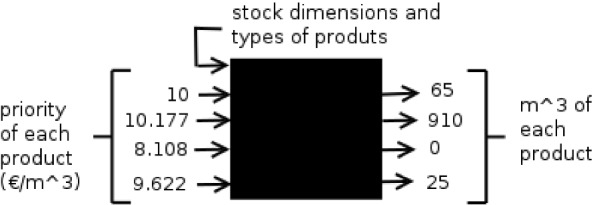
$\mathcal {DP}$ black box with an example of inputs and outputs

We denote the implementation of DP for this cutting problem as $\mathcal {DP}$. $\mathcal {DP}$ can be used as a black box with two inputs: the stock measurements and a priority vector. The output of the $\mathcal {DP}$ black box is the optimal amount of each product cut. The higher the value of a product in the priority vector, the higher tends to be the amount cut for such a product. Therefore, a simulation of the possible cuttings can be done by providing several different priority vectors as an input to the $\mathcal {DP}$ black box, since different amounts of products will be obtained. Then, the original meaning of the priority vector as monetary value is transformed into priorities of the products (even if internally, the $\mathcal {DP}$ black box implementation treats the priority vector as monetary values). See Fig. 2 for a visual representation of an example of use of the $\mathcal {DP}$ black box.

For the simulation phase, the $\mathcal {DP}$ black box is applied to each of the sampled pieces to find the most convenient priority vector. Recall that for the simulation phase, we only know the measurements of a sample of the stock. Besides, the $\mathcal {DP}$ black box is also used by each cutting machine in the field for performing the real cutting of many pieces with the priority vector selected in the simulation phase. Recall that the objective is to obtain the global targeted amounts of the products demanded by the customers. To achieve this, all the products from the cutting must be summed, then the priority vector can be adjusted according to the updated targeted amounts.

The vector should take into account that, for harvesting applications such as forestry, the cutting of the product is done on the field by many machines that locally use a priority vector in its own $\mathcal {DP}$ black box. The global counting of the total amounts already obtained at a certain time is done at a centralised point, and every cutting machine submits its counts to the central point. Then, total amounts are counted and sent to every machine, which can re-adjust their priority vector.

Ideally, the priority vector would be adjusted for each piece of real stock based on the counting of previous products cut. However, this is not possible for two reasons: firstly, it would be infeasible in real-life applications that have several thousand real raw material pieces (there are time limitations); second, it would be infeasible due to organisational issues (the system is centralised). Instead the stock is divided into subsets, and only a single priority vector can be applied to each stock subset. Either a subset is fully cut with a unique priority vector or none of its pieces is cut.

To obtain good solutions (consider that the re-adjustment of the priority vector is limited to the number of subsets, which is typically a relatively small number), a previous cutting simulation on the sampled stock should be done. The sampled stock is also divided into subsets (the sampling is proportional to the sizes of the stock subsets). After doing the simulation with several priority vectors generated, the most desirable priority vector is selected for every subset and it is used as an input of the machines that use the same $\mathcal {DP}$ black box to cut the real stock. Note that when the real cutting happens, the measures of each piece are known (since the machines measure the stock in the real field).

### Problem formalisation

In this subsection, we provide mathematical formulations and definitions for formalising the problem treated in this paper. Below, the reader can find a list of the notations used through the paper.

- ${\mathcal {K}}$ is the set of subsets of raw material pieces, where each subset $k\in {\mathcal {K}}$ has the following components:
  - *R* is the set of sampled pieces
  - *Q* is the complete set of pieces Therefore, $R \subset Q$ (generally *R* and *Q* are different for each subset but to simplify the description, here we assume that they are the same)
  - ${ \vec{\sigma }_r}$ contains the measurements of the sampled pieces
  - ${ \vec{\sigma }_q}$ contains the measurements of the whole set of pieces
- ${\mathcal {M}}$ is the set of product types, and we represent a type of product $m_j \in {\mathcal {M}}$ as a tuple $m_j=\langle s_j, z_j \rangle$, where:
  - $s_j\in {\mathbb {R}}$ is the size of a piece of $m_j$. Depending on the number of measurements analysed, $s_j$ can represent: lengths for 1-dimension (e.g. *m*), areas for 2-dimensions (e.g. $m^2$) or volumes for 3-dimensions (e.g. $m^3$).
  - $z_j$ is a vector of dimensions of $m_j$. For instance, if $m_j$ has the shape of a rectangle, $z_j$ would be the required length and width for $m_j$.
- $\vec{ \nu} \in \mathbb {Q_+}^{|{\mathcal {M}}|}$ denotes the priority vector, which is a vector of $|{\mathcal {M}}|$ continuous variables. Each $v_j$ represents the value associated to the type of product $m_j \in {\mathcal {M}}$ per unit of $s_j$. For instance, $v_j$ could represent cost per unit.
- ${\vec{p}}\in \mathbb {Q_+}^{|{\mathcal {M}}|}$ is a cutting instruction noted as ${\vec{p}}= \langle a_1, \dots , a_{|{\mathcal {M}}|} \rangle$, where $a_j$ represents the amount of units of product $m_j \in {\mathcal {M}}$ cut from certain subset of raw material pieces.

As previously mentioned, in the uncertain variant of our MSSCSP, it is only possible to have indirect control over the patterns via the priority vector. A vector of dimensions $\vec{\sigma }$ of |*R*|/|*Q*| of the raw material pieces and a set of product types ${\mathcal {M}}$ are provided to the $\mathcal {DP}$ implementation (as a black box) which uses the priority vector $\vec{ \nu}$ to calculate the corresponding amounts of products of each type ${\vec{p}}$ to be produced. This process is illustrated in Fig. 2. $\mathcal {DP}$ can be represented as the following mapping function:

1 $$\begin{aligned} \mathcal {DP}({\mathcal {M}},\langle \vec{\sigma }_1, \dots , \vec{\sigma }_{|R|/|Q|}\rangle , \vec{ \nu} ) \rightarrow {\vec{p}}. \end{aligned}$$

The $\mathcal {DP}$ algorithm simulates the cutting of each raw material piece to maximise its total value. This total value is obtained by summing the products of the units of each product cut by its associated value. Once this process is finished for all the raw material pieces, the sum of products represents a cutting instruction for the subset of raw material pieces. Here, we note that the greater the value of $v_i$ in a priority vector $\vec{ \nu}$ where other values are fixed, the greater (or equal) the units of product $m_i$ will be in its associated cutting instruction *p* after running algorithm $\mathcal {DP}$. The same occurs with the opposite situation: a lower or equal number of pieces are obtained when the values of $v_i$ are decreased.

To illustrate this, we present Example 1, in which there is an increase of units of the fourth type of product (there is also a decrease of units in other products). Even so, there is a saturation point at which the units cannot be increased any more. In this example, the saturation point of the fourth type of product has been reached. If we increase its associated value in the priority vector to more than 11.122, the same amount of product will be produced ($902 m^3$).

#### Example 1

Examples of priority vectors and their corresponding cutting instructions:

- $$\mathcal {DP}({\mathcal {M}},\vec{\sigma },\langle 10, 10.177, 8.108, \mathbf{9 .622} \rangle ) \rightarrow \langle 65, 910, 0, \mathbf{25} \rangle m^3$$
- $$\mathcal {DP}({\mathcal {M}},\vec{\sigma },\langle 10,10.177, 8.108, \mathbf{10 .122} \rangle ) \rightarrow \langle 51, 734, 0, \mathbf{216} \rangle m^3$$
- $$\mathcal {DP}({\mathcal {M}},\vec{\sigma },\langle 10,10.177, 8.108, \mathbf{11 .122} \rangle ) \rightarrow \langle 34,64,0, \mathbf{902} \rangle m^3$$
- $$\mathcal {DP}({\mathcal {M}},\vec{\sigma },\langle 10,10.177, 8.108, \mathbf{35} .\mathbf{5} \rangle ) \rightarrow \langle 34,64,0, \mathbf{902} \rangle m^3$$

### Vehicle routing problem

In our harvesting application, there are substantial costs associated with moving the cutting machines from one subset (or “block”) of stock to other blocks. The VRP, therefore, lends itself to the application as we need to design an efficient route through the forest. For the forestry harvesting the problem the blocks represent sub-areas of the forest and the cutting machines are harvesters.

Note that in the problem treated in this paper, there are several differences with the classical VRP. Here, the vehicles do not deliver to customers. Instead, they cut selected blocks of stock, placed them in different locations. In addition, they move all together from one block to another. Typically, the costs depend on the distance, because of energy resources (such as gasoline) required for such shifts.

**Fig. 3:**
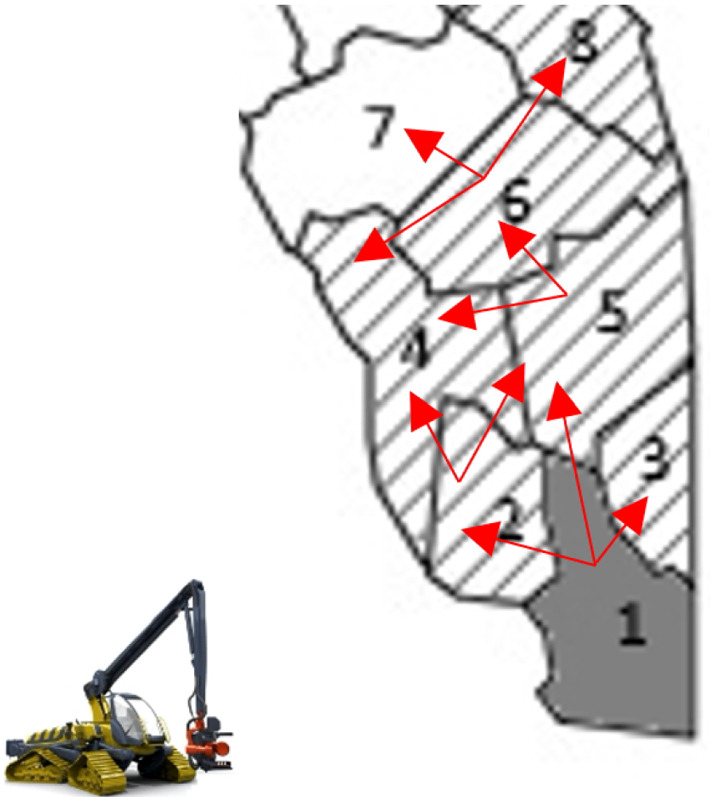
Possible contiguous routes with minimum costs, starting in the block 1

For the forestry harvesting problem, the shifts to non-neighbouring blocks are allowed because it might be more convenient to harvest certain blocks and leave unharvested neighbour blocks due to specific demands (for example, due to the specific types of products required and the characteristics of the logs of the specific block). In this case, because of the extra distance that the harvesters must traverse, there is a big extra cost associated with the shifts to non-neighbouring blocks as penalisation. For example, Fig. 3 shows the possibilities of routes for the cutting machines over contiguous blocks, which imply minimum costs for the shifts. The starting point is the dark-grey block. Harvested blocks are shaded, unharvested blocks are clear, and possible directions are shown with the red arrows. Note that sub-tour elimination constraints are still needed because in most of the cases, the solutions represent shifts to neighbouring blocks and, therefore, we have to avoid to re-visiting (and in this case, re-harvesting) the same block.

## ILP models

In this section, we introduce the ILPs for the static problem (ignoring uncertainties in the stock). In the following section (Sect. 5), we will present a reactive approach that incorporates such models and deals with the differences between the amount of stock expected, and the amount actually obtained. Recall that Sect. 3.3 explains the problem formalisation. A list of the additional notation used in this section of the paper is given as follows.

- $\vec{c}$ is a vector of costs. Each $c_k$ is the cost associated to the stock subset *k*.
- $\vec{d}$ is a vector of demands. Each $d_j$ is the demand for each type of product *j*.
- *E* is a matrix denoting the cost $e_{kl}$ of moving from block *k* to block *l*.
- $\vec{u}_{ik}$ represents the amounts of products cut from the block of stock *k* with the cutting instruction *i*.
- $\vec{ \nu} _{ik}$ is the priority vector associated with the cutting instruction *i* for the block of stock *k*.
- Variable $x_{ik}$ indicates whether stock subset *k* is cut with the cutting instruction *i*.
- Variable $y_{kl}$ indicates whether the harvester moves from the block *k* to the block *l*.

We would like to mention that, initially, a finite set of cutting instructions ($i=1 \ldots n$) must be precomputed for each sampled stock subset using the $\mathcal {DP}$ black box (see Fig. 2) to be able to solve these ILPs with standard optimisation software. It must also adequately cover all possible cutting instructions for each subset of sampled raw material pieces *k* (note that there are infinitely many possible priority vectors.) In Sect. 6, we use Monte Carlo simulation [see Mooney (1997)]. Thus, we generate a certain number of random priority vectors for each subset of raw material pieces $k\in {\mathcal {K}}$. Then, using the $\mathcal {DP}$ algorithm with such priority vectors [see Eq. 1], their corresponding cutting instructions are computed and stored in $\vec{u}_{ik}$ ($i=1 \ldots n$) (*n* is assumed to be the same for all the subsets to simplify the notation). Therefore,

2 $$\begin{aligned} \vec{u}_{ik} \leftarrow \mathcal {DP}: ({\mathcal {M}}, \langle \vec{\sigma }_1, \dots , \vec{\sigma }_{|R|}\rangle _k,\vec{ \nu} _{ik}) (\forall i,k). \end{aligned}$$

Then, $\vec{u}$ and its associated $\vec{ \nu}$ are provided as an input to Algorithm 1. There are also other input parameters: the set of types of products ${\mathcal {M}}$ and their demands $\vec{d}$, the set of stock subsets ${\mathcal {K}}$, their costs $\vec{c}$, and the real measurements of the pieces of each set ($\langle \vec{\sigma }_1, \dots , \vec{\sigma }_{|Q|}\rangle _k \forall k \in {\mathcal {K}}$). In the following, we describe an ILP that only handles the MSSCSP, then, subsequently, we present an ILP that handles the MSSCSP and also the VRP.

### ILP for the MSSCSP

In this section, we explain the ILP model from Prestwich et al. (2015) that handles the MSSCSP. We denote it as $h(\vec{d},\vec{u}, \vec{c}, {\mathcal {K}})$ and it is defined as follows:

3 $$\begin{aligned}&\text{ argmin } \sum _{i=1}^{n} \sum _{k=1}^{|{\mathcal {K}}|} c_{k} x_{ik},\\&\text{ s.t. } \nonumber \end{aligned}$$

4 $$\begin{aligned}&\sum _{i=1}^{n} \displaystyle \sum _{k=1}^{|{\mathcal {K}}|} u_{ikj} x_{ik} \ge d_j&\forall j \in {\mathcal {M}}, \end{aligned}$$

5 $$\begin{aligned}&\sum _{i=1}^{n} x_{ik} \le 1&\forall k \in {\mathcal {K}}, \end{aligned}$$

6 $$\begin{aligned}&x_{ik} \in \{0,1\}. \end{aligned}$$

This ILP model has decision variables $x_{ik}$ which indicate whether stock subset *k* is cut with the cutting instruction *i*. The objective is to minimise the total cost of the raw materials subsets used in the cutting process for satisfying the demands (the cost of each stock subset is denoted as $c_k$). Note that if a subset of raw materials pieces *k* is not used for satisfying the demands (and is, therefore, not cut) then all its decision variables ($x_{ik} \forall i \in n_k$) are zero. Note also that the constraint (4) ensures that the demands are fulfilled and the constraint (5) prevents the use of more than one cutting instruction in a subset of raw material pieces.

### ILP for the MSSCSP combined with the VRP

In the ILP model explained in Sect. 4.1, the stock subsets are selected randomly, without regard for the location of the subsets. However, as mentioned in Sect. 3.4, in real-life applications, there are typically some costs associated with shifting the cutting machines between different locations at which the subsets of stock are located. To model this characteristic, we modify $h(\vec{d},\vec{u}, \vec{c}, {\mathcal {K}})$ to include neighbourhood and routing costs, and denote this combined Cutting Uncertain Stock and Vehicle Routing Problem by $s(\vec{d},\vec{u},\vec{c}, {\mathcal {K}}, E)$ where *E* is a matrix denoting the cost $e_{kl}$ of moving from block *k* to block *l*. We also include the additional decision variable $y_{kl}$ which indicates whether the harvester moves from the block *k* to the block *l*.

The first term of the objective function (7) reflects the cost of harvesting a forest block, while the second term describes the cost of the route followed by the harvester. Typically, contiguous blocks have a minimal cost of traversal, while non-contiguous blocks have high travel costs between them. We also include a dummy subset to the stock subsets (at $k = 0$), which acts as the depot in the classic VRP.

Constraint (8) is the original demand constraint, while the rest of the constraints differ from the original ILP model. Constraints (9) and (10) are the bridging constraints that link the CSP to the VRP. They ensure that if a block is visited, then it must be cut. Constraints (11) and (12) state that there are only a single entrance and a single departure from a block (then, blocks cannot be revisited in the path). The summation in (12) begins from $l=1$ as the harvester is not allowed to move backwards to the block it has just visited. Constraint (13) is a flow conservation constraint that ensures the continuity of the path. As we may allow the path to be discontinuous, we use $\le 1$ instead of $=0$. Constraint (14) ensures that the harvester only leaves the depot once. Although more effective formulations such as those in Laporte (1992) exist, we have used the Miller–Tucker–Zemlin sub-tour elimination constraints in (15) for simplicity. Constraint (16) eliminates trips from a block to itself. Finally, in the lines (17) and (18), the binary decision variables are defined. Our version of the VRP is incapacitated, as there is no limit to how much the harvester can cut:

7 $$\begin{aligned}&\text{ min } \sum _{i=1}^{n} \sum _{k=1}^{|{\mathcal {K}}|} c_k x_{ik} + \sum _{k=0}^{|{\mathcal {K}}|} \sum _{l=0}^{|{\mathcal {K}}|} e_{kl} y_{kl},\\&\text{ s.t. } \nonumber \end{aligned}$$

8 $$\begin{aligned}&\sum _{i=1}^{n} \sum _{k=1}^{|{\mathcal {K}}|} u_{ikj} x_{ik} \ge d_j&\forall j \in {\mathcal {M}}, \end{aligned}$$

9 $$\begin{aligned}&\sum _{k=0}^{|{\mathcal {K}}|} y_{kl} = \sum _{i=1}^{n} x_{il}&\forall l \in {\mathcal {K}} \setminus \{0\}, \end{aligned}$$

10 $$\begin{aligned}&\sum _{l=0}^{|{\mathcal {K}}|} y_{kl} = \sum _{i=1}^{n} x_{ik}&\forall k \in {\mathcal {K}} \setminus \{0\}, \end{aligned}$$

11 $$\begin{aligned}&\sum _{k=0}^{|{\mathcal {K}}|} y_{kl} \le 1&\forall l \in {\mathcal {K}} \setminus \{0\}, \end{aligned}$$

12 $$\begin{aligned}&\sum _{l=1}^{|{\mathcal {K}}|} y_{kl} \le 1&\forall k \in {\mathcal {K}}, \end{aligned}$$

13 $$\begin{aligned}&\sum _{k=0}^{\left|{\mathcal {K}}\right|} y_{kv} - \sum _{l=1}^{\left|{\mathcal {K}}\right| } y_{vl} \le 1&\forall v \in|{\mathcal {K}}| \setminus \{0\}, \end{aligned}$$

14 $$\begin{aligned}&\sum _{l=1}^{|{\mathcal {K}}|} y_{0l} = 1, \end{aligned}$$

15 $$\begin{aligned}&w_k - w_l + |{\mathcal {K}}| \times y_{kl} \le |{\mathcal {K}}|-1&\forall k,l \in {\mathcal {K}} \setminus \{0\}, \end{aligned}$$

16 $$\begin{aligned}&y_{kk} = 0&\forall k \in {\mathcal {K}}, \end{aligned}$$

17 $$\begin{aligned}&x_{ik} \in \{0,1\}&\forall i, k , \end{aligned}$$

18 $$\begin{aligned}&y_{kl} \in \{0,1\}&\forall k, l. \end{aligned}$$

To reduce the computational time, we use a Rolling Horizon approach (Peeta and Mahmassani 1995) to solve the problem. A rolling horizon approach divides the problem into a series of time-periods. The current time-period is modelled precisely, while the rest of the time-periods are aggregated and solved using a relaxed model. This approach has been shown to produce close-optimal solutions to challenging, real-world logistics problems while significantly reducing their computation times (Marquant et al. 2015; Zhan et al. 2016). We implement this approach in the following way. For the blocks that are not directly connected to the depot (i.e. they cannot be reached by the harvester in this time-period), we relax the restriction that they must be harvested using only one cutting instruction. We remove the binary restriction of (17) and allow $x_{ik}$ to take continuous values (19), thus speeding up the computation process. To achieve this, we define binary variables $b_{ik}$, so that when there is a connection from the depot to a block *k* (i.e. when $e_{0k} = 1$), we constrain $x_{ik}$ to be equal to $b_{ik}$. The optimisation problem is, therefore, modified as

19 $$\begin{aligned}&x_{ik} \in [0,1]&\forall i, k , \end{aligned}$$

20 $$\begin{aligned}&b_{ik} \in \{0,1\}&\forall i, k , \end{aligned}$$

21 $$\begin{aligned}&x_{ik} = b_{ik}&\text{ if } e_{0k} = 1, \end{aligned}$$

22 $$\begin{aligned}&\sum _{i=1}^n b_{ik} = 1&\forall k \in {\mathcal {K}}, \end{aligned}$$

where (17) has been replaced by (19), and (20), (21) and (22) have been added. In this way, the full ILP is solved only for the very few blocks connected to the depot. This approach is applied again and again in subsequent iterations until the harvesting process is complete.

## Dealing with uncertainties

In the above-mentioned ILPs, the differences between the expected cut products and the real cut products are not considered. In this section, we present a reactive approach that dynamically re-calculates the amounts targeted after cutting each real stock subset, and re-computes new solutions for the rest of the cutting process. This approach can work both with the ILP that only solves the MSSCSP, as well as with the ILP that also handles the VRP. We would like to reiterate that this algorithm is applied on-line, since the measurements of most of the raw material pieces are only known once the real cutting process is being performed. In addition, the real amounts of types of products obtained for each stock subset are only known after a stock subset is cut.

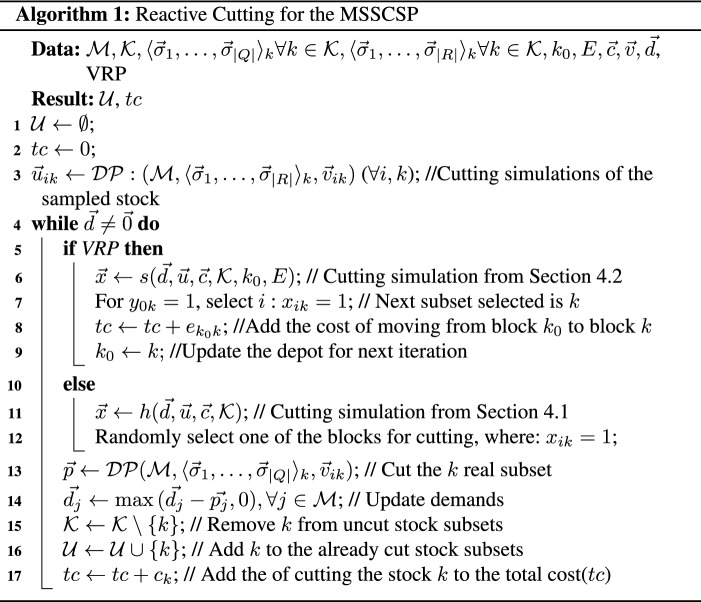



In the following, we describe the reactive algorithm presented in this paper, Algorithm 1. A list of the additional notation used in this section is given as follows:

- $k_0$ is the depot.
- *E* is the matrix of transportation costs between blocks.
- VRP is a Boolean variable specifying if the problem is a VRP or not.
- ${\mathcal {U}}$ stores the stock subsets that have already been cut for satisfying the given demands.
- *tc* keeps track of the total cost of the cutting process: the costs of the stock blocks selected for cutting combined with the costs of moving the cutting machines.

The input parameters of Algorithm 1 are the parameters described in Sect. 3.3: the set of products types (${\mathcal {M}}$), the set of subsets of raw material pieces (${\mathcal {K}}$), with the associated measurements of the sampled and real stock ($\langle \vec{\sigma }_1, \dots , \vec{\sigma }_{|R|}\rangle _k$ $\forall k\in {\mathcal {K}}$ and $\langle \vec{\sigma }_1, \dots , \vec{\sigma }_{|Q|}\rangle _k \forall k\in {\mathcal {K}}$, respectively), the depot ($k_0$), the matrix of transportation costs from blocks *E*, the costs associated to the cutting of each subset $\vec{c}$, the original priority vector $\vec{ \nu}$, the demands $\vec{d}$ and a Boolean variable specifying if the problem is a VRP (denoted as *VRP*). The output parameters of the Algorithm 1 are ${\mathcal {U}}$ (stock subsets that have already been cut) and *tc* (associated total cost of such cutting).

Algorithm 1 first initialises ${\mathcal {U}}$ and *tc* (see lines 1 and 2). Both variables are returned by the algorithm once the demands of all the types of products are fully satisfied (stopping condition of the loop, in the line 4). In line 3, before the start of the iterations, the algorithm performs Monte Carlo simulation [see Mooney (1997)] of all the sampled subsets of stocks, which returns $\vec{u}_{ik}$ (amounts obtained for all the types of products each stock subset *k* for each cutting instruction *i* simulated).

In each iteration, the algorithm first solves the ILP model (with/without VRP, lines 6 and 11 respectively) which returns the optimal stock subsets to cut with the selected general cutting instructions (according to the sampled stock). After this simulation process, the algorithm either randomly selects one of those stock subsets (without VRP, line 12), or it selects a neighbouring stock subset. The selected neighbour is the best of those that are next to the depot (with VRP, line 7). In the latter case (with VRP), line 8 adds the cost of shifting the cutting machines from certain block to the next block of stock selected for cutting. In the line 9, the depot for the next iteration is fixed to to the current block of stock selected for the cutting in the current iteration (because the cutting machines will start their path in such block).

Subsequently, the algorithm performs the real cutting of the real stock subset with the vector of values of the cutting instruction (line 13), both selected in the simulation phase. The following steps are responsible for updating: (i) the targeted demands (by subtracting the amounts obtained from the cutting, in line 14), (ii) the status of the selected stock subset from uncut to already cut (lines 15 and 16) and (iii) the total cost (by adding the stock subset cost, in line 17). We would like to mention that sometimes the customers might change their demands of the different products over time or there might be new demands from other customers (likely to happen in real applications). Note that our dynamic algorithm can accommodate these modifications over the demands of types of products (while static ILP approaches cannot). This could be done by updating $\vec{d}$ at any step of Algorithm 1.

## Evaluation

In this section, we compare our reactive algorithm (with and without VRP) with another dynamic approach from the literature: the Simulated Annealing Like Algorithm (SALA) metaheuristic from Murphy et al. (2006). The motivation for the selection of this algorithm is provided in Sect. 2. This metaheuristic has been very popular in the forestry harvesting industry (which is a clear example of the uncertain MSSCSP). This metaheuristic approach makes local changes in the priority vector iteratively to obtain better cutting instructions (with less waste of resources). In this section, we present experiments performed with real forestry instances (provided by our industrial partner). We focus on minimising the waste of natural resources which leads to a very attractive aspect for these industries: an increase in revenue.

In the following, we describe some details associated with forestry harvesting. In the forestry harvesting problem, the logs of the trees (stock) must be cut into smaller log-pieces (types of product) by harvesting machines to satisfy the demands of the customers. The log-pieces have shape specifications (minimum diameter, length and so on) and their volumes are measured typically in cubic metres (m$^3$).

The forest is divided into several areas, each with a given specific cost. The measurements of the sampled trees are derived from 3*D* scans. Note that it would be too costly to measure the measurements of all the trees, as there are possibly several thousand in a forest. As previously mentioned, the objective function is to minimise the total cost associated with the subsets harvested. This typically implies the selection of low-value blocks, as this ensures that the best trees of the forest are left intact. As previously mentioned, only some stock subsets are selected for satisfying the demands, and therefore, only this selection of the forest is harvested. Note that the forest owners want to extract the maximum benefit from a selling, which for a given demand is obtained by harvesting the stock subsets with the total minimum cost. The rest of the stock subsets can be used in future demands.

Both the original metaheuristic algorithm of Murphy et al. (2006) and the algorithm presented in this paper (Algorithm 1) were implemented in Java. The evaluation was done on a 2.3 GHz Intel Core i7-4850HQ processor with 16 GB of RAM. Our industrial partner also provided us with a black box software that carries out the dynamic-programming-based ($\mathcal {DP}$) simulation that implements $\mathcal {DP}$ (see Eq. 1), which is used by the two approaches. Since DP is an exact/complete algorithm, other DP implementations, such as the one in Pnevmaticos and Mann (1972), could have been used for $\mathcal {DP}$ with equivalent results. Therefore, these experiments are reproducible. The ILP models of Algorithm 1 were solved with CPLEX 12.3 solver with a time cutoff of 90 s and fixing as termination criterion an ILP gap of 0.025 (corresponding to $2.5\%$).

In this section, we have analysed a real forestry instance that represents a forest for which the total volume of logs is $4640.66\text {m}^3$. However, for solving the problem, the measurements of only $25\%$ of trees (rounded to the nearest integer) of each subset were known. Then, the total volume of the sampled logs of such forest is $1145.15\text {m}^3$. The forest is composed of 26 subsets whose volumes are in the interval $[84.3, 293] \text {m}^3$. The costs of the individual subsets are in the interval €[4315.25, 20, 422.16] and the total cost of all them is €306, 295.23. We would like to mention that for this instance the costs of the subsets were determined by applying the dynamic-programming-based ($\mathcal {DP}$) simulator to them with an input priority vector equal to the prices of the log-pieces in the open market (typically those prices increase according to the volume of the log-pieces types).

### Reactive approach without vehicle routing

**Fig. 4:**
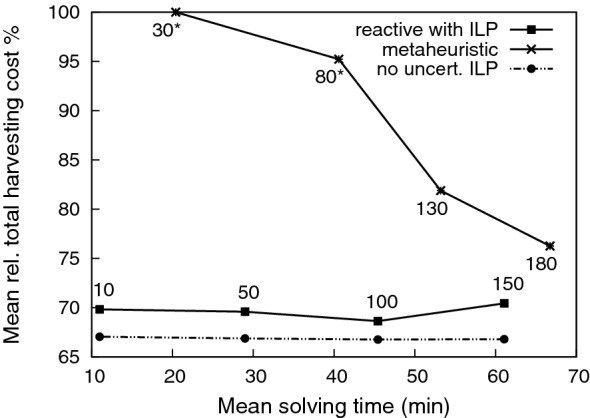
Quality of the solutions obtained by the two reactive approaches and the ILP that does not consider uncertainty

In this section, we evaluate the MSSCSP without the VRP component. Figure 4 shows the mean results of 20 runs for each experiment performed with seven types of log-pieces ($|{\mathcal {M}}|=7$). The demands of each log-piece were randomly selected in the interval $[200,600]\text {m}^3$. We performed 20 runs because both approaches stochastically select the next subset to cut (which affects the quality of the solutions obtained). For Algorithm 1, the next subset to cut is randomly selected over the best uncut subsets selected by solving the current ILP model. However, for Murphy et al. (2006), the next subset to cut is randomly selected over all the uncut subsets (because this algorithm does not select specific subsets to cut, instead it chooses them randomly). Furthermore, as explained below, there exists more stochasticity in the algorithms. In our technique, the cutting instructions are randomly generated via Monte Carlo algorithm. Hence, we have re-computed them in each of the 20 runs.

Figure 4 shows the quality of the solutions obtained in the vertical axis, that is the mean relative percentage of the total cost of the harvested stock subsets (the percentage of the total cost of the subsets used for the harvesting over all the total cost of all the subsets). Note that this total cost is averaged for each of the 20 instances with generated random demands. We also would like to point out that the lower this percentage is, the greater the solution quality. The mean solving time in minutes is represented on the horizontal axis. For our technique, this means the sum of the cutting instructions generation and the time consumed by Algorithm 1. Each specific number associated to the points of our technique represents the number of cutting instructions generated for each stock subset (e.g 100 cutting instructions for each of the 26 subsets, which makes a total of 2600 cutting instructions generated for a single run). Each specific number associated to the points of the metaheuristic represents its cutoff time (in seconds) for each stock subset (e.g. for each subset local changes are made during 80 s and the best combination is kept).

Figure 4 also shows the “static” scenario (dashed line with circles), where there are no uncertainties about the stock: what would be the harvesting cost if the sampled stock (known data) were the whole population (rather than 25%)? Note that in this scenario there would be no need for a dynamic approach. To calculate values, the ILP model was run only once with the sampled data. We would like to note that this “static” way of solving the problem is very similar to those in Prestwich et al. (2015) and Dems et al. (2017). However, real-life applications typically have associated uncertainty which it is unrealistic to ignore. We did not include such an approach for comparison purposes (because it does not consider the same uncertain scenario as the other approaches so the comparison would be unfair) but to show how well our dynamic approach (solid line with squares) behaves with the uncertain scenario. Obviously, the closer the results of an approach are to the results of the scenario without uncertainty, it means that the approach deals better with the uncertainty. Note that our approach has less than 5% difference from the scenario without uncertainty, while the metaheuristic is quite far from that scenario, especially for low computation times.

It can also be observed that the metaheuristic algorithm behaves poorly unless the cutoff time is very high. It needs a significant amount of time to find a good combination, which increases when the number of product types grows. The $*$ over the 30 s and 80 s cutoffs indicates that the metaheuristic could not find a solution for some instances (all the forest was harvested but the demands were not satisfied). Specifically, for 30 s, no solution was found for any of the runs). Our solutions have a lower cost of more than $6.4\%$ than the other solutions obtained by the metaheuristic during 66 min (even than our solutions obtained in 11 min). This represents an increase in the benefit of this instance of at least €19, 602.9 and a huge improvement in computation time. One reason for this high performance is that we consider the stock subsets selection as an optimality criterion, while the metaheuristic does not. The other reason is that metaheuristics are incomplete/inexact algorithms which do not guarantee to find a globally optimal solution, unlike complete/exact algorithms. Metaheuristics sample a subset of solutions from the search space, while complete/exact algorithms explore completely the search space.

Figure 4 also suggests that the quality of our solutions barely improves when the number of generated cutting instructions increases. For this reason, the generation of a high number of cutting instructions is not recommended in time-sensitive applications. Again, we emphasise the economical benefits of our technique over the metaheuristic, especially considering that in many real applications of this type decisions must be made quickly (on-line cutting processes), such as with the forestry harvesting problem.

### Reactive approach with vehicle routing

In this section, we evaluate our reactive technique, which includes the ILP model that also considers the VRP, where the vehicles are the cutting machines (in this specific case they are harvesters). Note that the metaheuristic algorithm does not deal with the VRP; nevertheless, we compare our solutions with VRP with the metaheuristic solutions from the previous section (even if it is not a fair evaluation because costs of moving over the blocks are not considered for the metaheuristic algorithm). The same problem with 26 of blocks is first analysed, then a larger problem with more blocks is evaluated.

#### Routing through 26 blocks

**Fig. 5:**
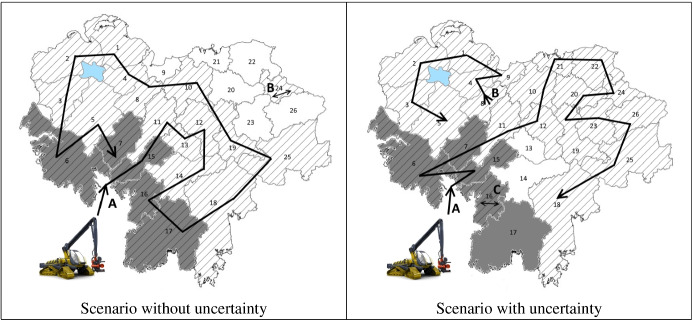
Path of the harvesters through the forest

The same instance (volume, types of logs, demands, etc.) as in Sect. 6.1 was analysed. We set the cost of moving the harvesting machines between neighbouring blocks to €0, while the cost between non-neighbouring blocks was set to €10, 000. However, the costs could also depend on the distance between blocks or other criteria given by the real application. Figure 5 shows a map of the forest with the representation of the neighbourhood of the blocks of stock. It should be noted that since the real neighbourhood information was not provided by our industrial partners, we randomly assigned neighbours to the blocks. The dark-coloured blocks indicate which blocks are connected to the road where the harvesting machines can access to the forest. Thus, the depot is connected to blocks 6, 7, 15, 16 and 17.

To evaluate our technique (described in Sect. 4.2), we generated 30 cutting instructions for each of two scenarios: with and without uncertainty (Fig. 5). For the scenario without uncertainty, we assume that the characteristics of all the stock are known a priori. For the scenario with uncertainty, only a sample of the real stock is known (as in Sect. 6.1, we use a sample of $25\%$ of trees). Therefore, it is expected that this uncertainty will affect the routing of the harvester. In both figures, we can observe the route taken by the harvester. The shaded blocks are the ones harvested. Each solution was found in less than 2 min. We would like to point out the large difference in computation time compared to the approaches evaluated in Sect. 6.1. This is mainly due to the relaxation of the model over the blocks that are not connected to the current location of the harvesting machines (this relaxation is described in Sect. 4.2).

First we analysed the scenario without uncertainty (left panel of Fig. 5), for which we obtained a percentage mean relative total harvesting cost of $70.85\%$. While this percentage is more than that obtained by our reactive approach without VRP, it is still less than the one obtained by the metaheuristic of Murphy et al. (2006) (see Fig. 4). This increase in the percentage of blocks harvested over our technique without VRP is because routing costs restrict the freedom with which we can select blocks (even so, this scenario is the most realistic for many industries, such as forestry harvesting). It can be seen that in this case of no uncertainty, the harvester mostly follows one path, with a jump to block 24 at the end (which adds €10,000 to the total cost). The block 24 is selected because its low cost, plus the extra cost of moving the harvesters there, is cheaper than selecting a neighbouring block.

The right panel of Fig. 5 shows the scenario with uncertainty, i.e. the case in which the sampled data represent a small portion of the whole real data (which is the typical scenario in real-life applications). Here, the percentage mean relative total harvesting cost is $86\%$. Due to the uncertainty, the algorithm has a difficult time selecting a single path to follow through the forest, resulting in 3 different paths (A, B and C). This results in an increase in the cost due to the non-contiguous blocks selected. Even so, our technique performed performed better than the metaheuristic algorithm (with a solving time up to 45 min), see Fig. 4. Note that the solving time of our technique was less than 2 min.

#### Routing through 100 blocks

A larger problem instance with 100 blocks was solved by our technique for three different random demand scenarios of seven different types of products (see Table 1). For this large instance, to reduce the computation time, we implement a “search window” such that we only consider the 30 blocks closest to the depot in each iteration of the algorithm.

**Table 1:** Demands (m$$^3$$)

Log type	1	2	3	4	5	6	7
Demand 1	50	34	25	51	24	52	60
Demand 2	353	239	178	360	165	361	419
Demand 3	433	572	369	358	490	332	478

In Table 2, there are two columns for each demand evaluated: one with uncertainty and another without uncertainty. We observe that the discrepancy between the amounts expected from sampling and the amounts obtained leads to an increase in the number of blocks harvested. It also leads to an increase in the total harvesting cost, which illustrates the negative effect of having bad samples.

**Fig. 6:**
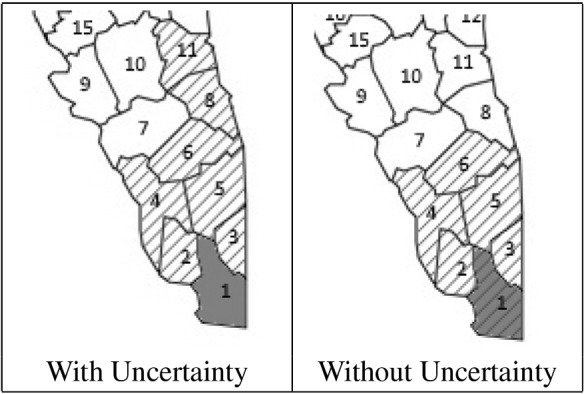
Blocks selected for harvesting for 100 blocks for demand 1

**Fig. 7:**
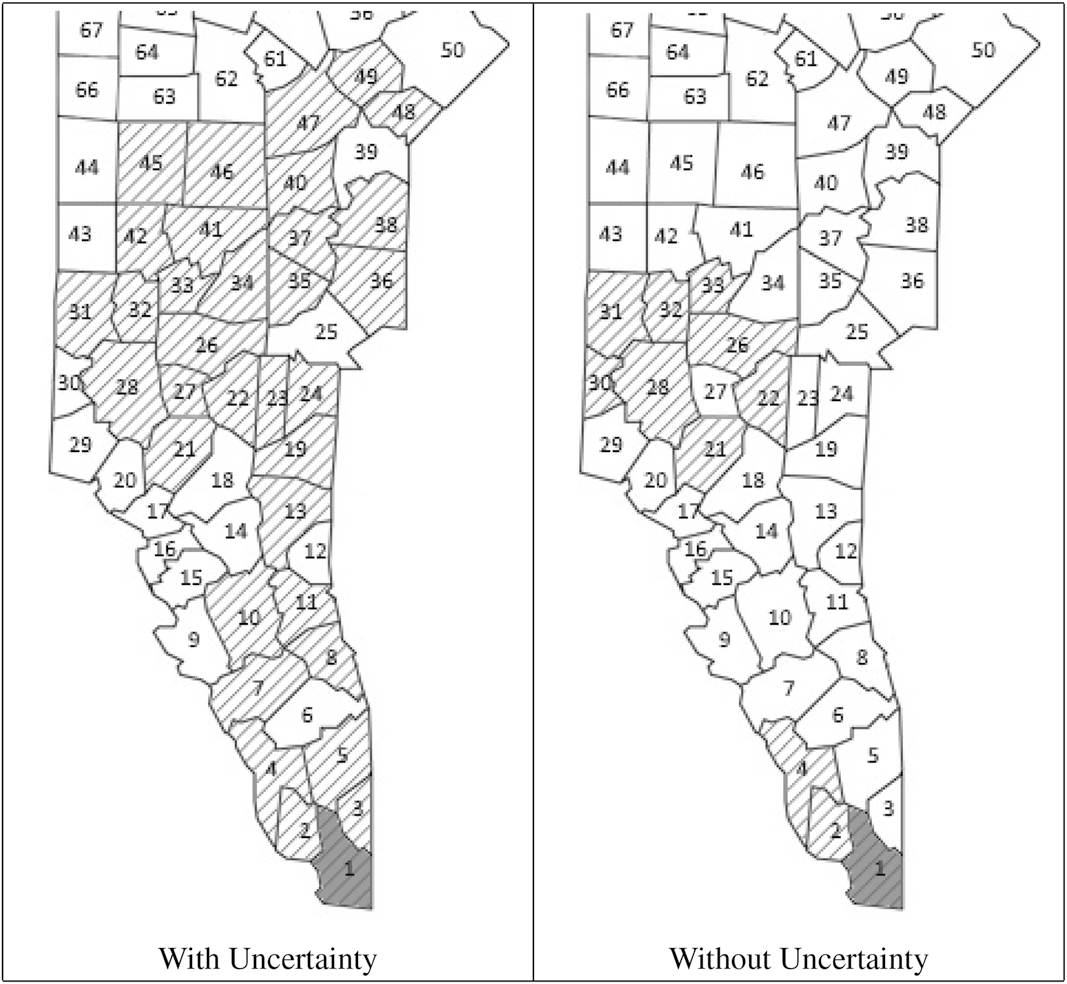
Blocks selected for harvesting for 100 blocks for demand 2

**Fig. 8:**
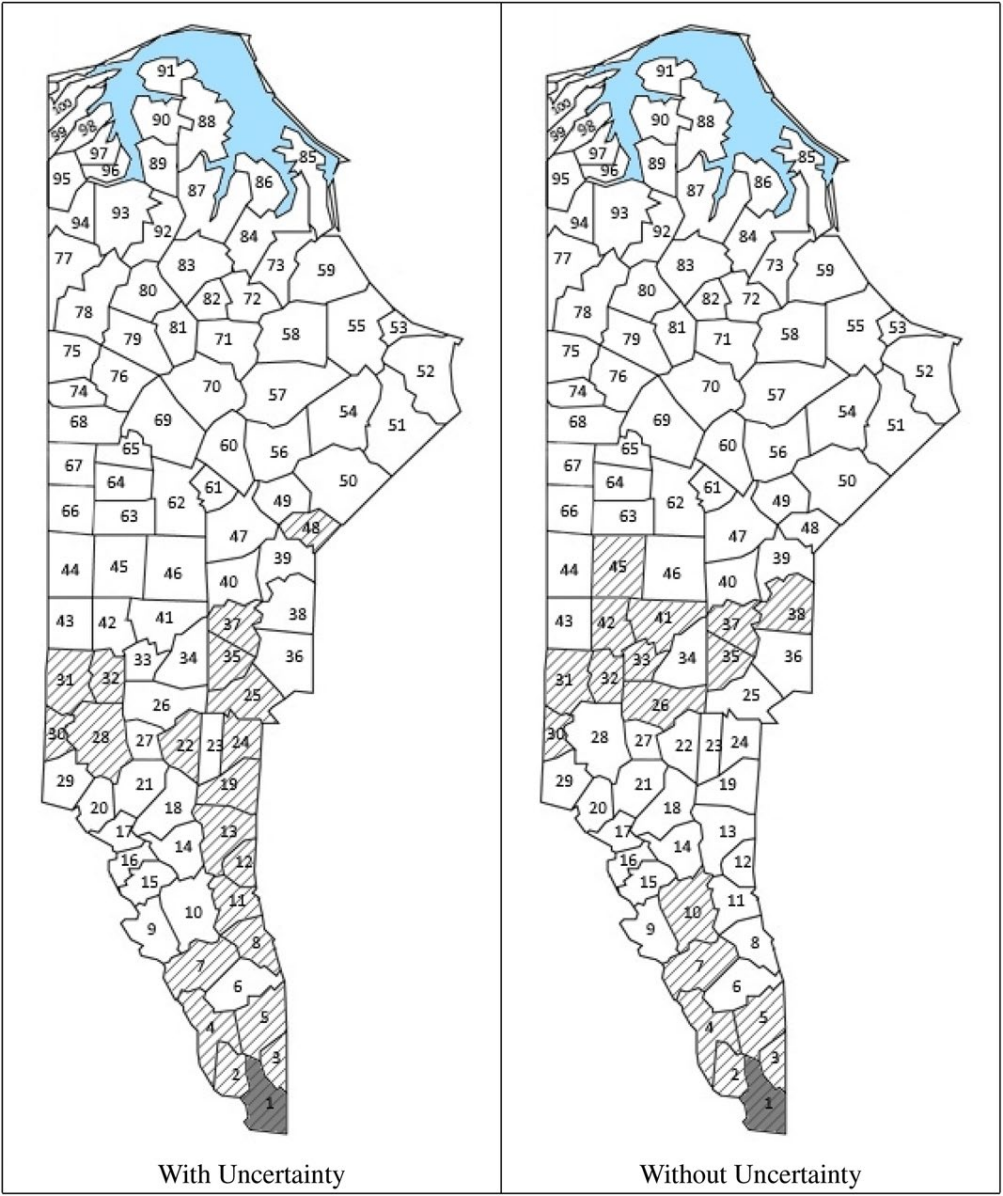
Blocks selected for harvesting for 100 blocks for demand 3

Figures 6, 7 and 8 show the blocks selected for harvesting for each demand scenario. In general, the blocks selected are seen to be contiguous. In certain cases, however, there are two or three groups of discontiguous blocks selected (Fig. 8). In demand scenario 2, the path produced by the algorithm leads the harvester into a group of low-yield blocks. This combined with the uncertainty results in a high number of blocks being selected for harvesting. As expected, when there is uncertainty, more blocks are harvested because the samples do not always accurately reflect the capacity of the forest.

**Table 2:** Results

Urcentainty	Demand 1		Demand 2		Demand 3	
	With	Without	With	Without	With	Without
Total cost (€)	58,265	49,361	325,133	110,532	276,491	158,011
Number of blocks harvested	7	6	34	11	20	18
Total solution time (s)	38	3	661	130	728	361

## Conclusions

In this paper, we have contributed to the literature by presenting the first reactive approach that uses ILP for solving a variant of the MSSCSP with uncertain stock and its combination with the VRP. The main extra difficulty of this variant lies in the fact that only the measurements of some samples of the real stock are known. This uncertainty ensures that the amounts obtained after harvesting will certainly be different from the amounts expected. This type of problem is composed of several stock subsets, and it is only possible to compute the amounts of products obtained after the cutting of each real stock subset. This fact motivated the development of a reactive approach that dynamically re-computes solutions after re-calculating the targeted amounts by solving the ILP model. Furthermore, our technique also selects the best stock subsets to cut given the current state of the targeted amounts. For this matter, we also present a novel ILP model that combines the MSSCSP with the VRP, in which the order of the blocks selected is computed based on neighbourhood costs.

This variant of MSSCSP will occur in real-life applications in which only some samples of the measurements of the stock can be taken by the industry. A very good example of such type of problems is population harvesting problems. In this paper, we evaluate a forestry harvesting problem with our technique and another dynamic approach: the popular metaheuristic of Murphy et al. (2006). This evaluation shows the greater performance of our techniques over the metaheuristic, especially for low and intermediate cutoff times. This fact is of great importance to the industry since in many real applications the cutting decisions are made on-line (therefore, they must be made quickly). Besides, our solutions typically provide paths for the cutting machines that are contiguous and, therefore, minimise the energy expended in the shifts, minimising then the total cost of cutting plus routing. Better solutions not only imply minimising resources used (sustainability target) but also an increase in the benefits for the industry.

In future work, we will focus on the combination of the MSSCSP with the VRP by modifying the travel costs to take into account the real distances between all stock subsets (blocks in the case of forestry) and obtaining real location data for the blocks. We also intend to investigate means of improving the accuracy of the sampled data, which should have a positive effect on the results in cases in which the sample data is not representative enough.
